# A practical guide to the implementation of AI in orthopaedic research – part 1: opportunities in clinical application and overcoming existing challenges

**DOI:** 10.1186/s40634-023-00683-z

**Published:** 2023-11-16

**Authors:** Bálint Zsidai, Ann-Sophie Hilkert, Janina Kaarre, Eric Narup, Eric Hamrin Senorski, Alberto Grassi, Christophe Ley, Umile Giuseppe Longo, Elmar Herbst, Michael T. Hirschmann, Sebastian Kopf, Romain Seil, Thomas Tischer, Kristian Samuelsson, Robert Feldt, ESSKA Artificial Intelligence Working Group

**Affiliations:** 1 Sahlgrenska Sports Medicine Center, Gothenburg, Sweden; 2 Department of Orthopaedics, Institute of Clinical Sciences, Sahlgrenska Academy, University of Gothenburg, Gothenburg, Sweden; 3 Department of Computer Science and Engineering, Chalmers University of Technology, Gothenburg, Sweden; 4 Medfield Diagnostics AB, Gothenburg, Sweden; 5 Department of Orthopaedic Surgery, UPMC Freddie Fu Sports Medicine Center, University of Pittsburgh, Pittsburgh, USA; 6 Department of Health and Rehabilitation, Institute of Neuroscience and Physiology, Sahlgrenska Academy, University of Gothenburg, Gothenburg, Sweden; 7 Sportrehab Sports Medicine Clinic, Gothenburg, Sweden; 8 IIa Clinica Ortopedica E Traumatologica, IRCCS Istituto Ortopedico Rizzoli, Bologna, Italy; 9 Department of Mathematics, University of Luxembourg, Esch-Sur-Alzette, Luxembourg; 10 Department of Orthopaedic and Trauma Surgery, Campus Bio-Medico University, Rome, Italy; 11 Department of Trauma, Hand and Reconstructive Surgery, University Hospital Münster, Münster, Germany; 12 Department of Orthopedic Surgery and Traumatology, Head Knee Surgery and DKF Head of Research, Kantonsspital Baselland, 4101 Bruderholz, Switzerland; 13 Center of Orthopaedics and Traumatology, University Hospital Brandenburg a.d.H., Brandenburg Medical School Theodor Fontane, 14770 Brandenburg a.d.H., Germany; 14 Faculty of Health Sciences Brandenburg, Brandenburg Medical School Theodor Fontane, 14770 Brandenburg a.d.H., Germany; 15 Department of Orthopaedic Surgery, Centre Hospitalier Luxembourg and Luxembourg Institute of Health, Luxembourg, Luxembourg; 16 Clinic for Orthopaedics and Trauma Surgery, Malteser Waldkrankenhaus St. Marien, Erlangen, Germany; 17 Department of Orthopaedics, Sahlgrenska University Hospital, Mölndal, Sweden

**Keywords:** Artificial intelligence, AI, Machine learning, ML, Large language models, Ethics, Explainability, Decision support systems, Digital twins, Provenance, Generalizability, Learning series, Orthopaedics, Research methods

## Abstract

Artificial intelligence (AI) has the potential to transform medical research by improving disease diagnosis, clinical decision-making, and outcome prediction. Despite the rapid adoption of AI and machine learning (ML) in other domains and industry, deployment in medical research and clinical practice poses several challenges due to the inherent characteristics and barriers of the healthcare sector. Therefore, researchers aiming to perform AI-intensive studies require a fundamental understanding of the key concepts, biases, and clinical safety concerns associated with the use of AI. Through the analysis of large, multimodal datasets, AI has the potential to revolutionize orthopaedic research, with new insights regarding the optimal diagnosis and management of patients affected musculoskeletal injury and disease. The article is the first in a series introducing fundamental concepts and best practices to guide healthcare professionals and researcher interested in performing AI-intensive orthopaedic research studies. The vast potential of AI in orthopaedics is illustrated through examples involving disease- or injury-specific outcome prediction, medical image analysis, clinical decision support systems and digital twin technology. Furthermore, it is essential to address the role of human involvement in training unbiased, generalizable AI models, their explainability in high-risk clinical settings and the implementation of expert oversight and clinical safety measures for failure. In conclusion, the opportunities and challenges of AI in medicine are presented to ensure the safe and ethical deployment of AI models for orthopaedic research and clinical application.

**Level of evidence** IV

## Introduction

**Fig. 1:**
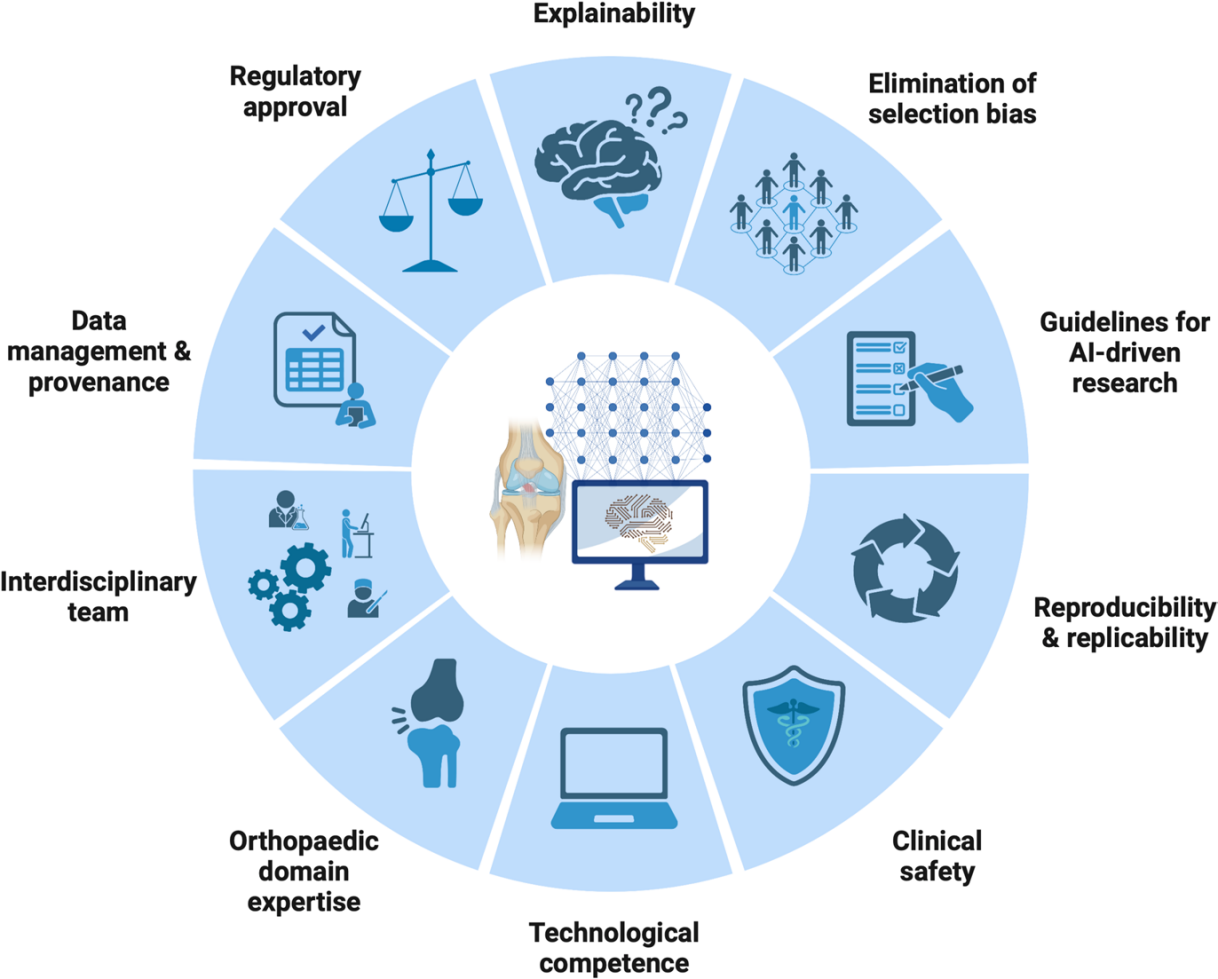
Challenges for the implementation of AI in orthopaedic research and clinical practice

Artificial intelligence (AI) is set to transform the landscape of medical research with innovative approaches to improve disease detection, clinical decision-making, and outcome prediction. The majority of medical research conducted throughout 20^th^ and early twenty-first centuries relied on well-established statistical methods for data analysis. However, increasingly sophisticated applications in engineering, business, and industrial sectors have shown the rapid technological advancement and maturity of AI, with a growing interest for implementation in medical research, and the healthcare sector [5, 37]. According to a conceptual framework developed by Autor and Levy the nature of work-related tasks can be classified as “routine” or “non-routine” and “cognitive” or “manual” [3]. Application of the same framework to tasks performed in clinical medicine and medical research can help a range of stakeholders to frame the transformative impact of digitalization and automation with AI-intensive technology in terms of the type of task performed (Table 1). As an example, preoperative planning for complex knee ligament surgery is a routinely performed cognitive task, which relies on the interpretation of physical examination findings, the results of diagnostic imaging, and choosing the operative approaches and techniques that are most suitable for the individual anatomy and lifestyle demands of the patient. This scenario is likely to benefit from the application of AI systems to facilitate steps involved in preoperative planning based on a set of patient- and surgery-related parameters. While the opportunities to harness the potential of AI in medicine are countless, the healthcare environment possesses several inherent characteristics and barriers to the adoption of AI for research, and clinical use. To ensure the effective and safe implementation of AI in medical research, proficiency with key concepts and terms related to AI-driven innovation, potential sources of bias and clinical safety are essential [12, 31]. The aim of this article is to introduce the opportunities and challenges in AI-intensive medical research to the orthopaedic research community, and to familiarize the reader with key terms and concepts illustrating current barriers to ethical and reliable implementation (Fig. 1). Additionally, the current article outlines a roadmap for subsequent sections of this learning series on the adaptation of AI to orthopaedic research. The current article is the first of a series of texts aimed at providing readers with the tools and best-practices to develop well-functioning AI systems with applications in orthopaedics, and focuses on the importance of ethical considerations, trustworthiness and the quality of AI-intensive research. A comprehensive technical introduction of AI to orthopaedic researchers will be provided in the forthcoming article.

**Table 1:** Examples of the transformative impact of AI-intensive applications across tasks performed in clinical medicine and medical research

**Task category**	**Routine** (Repeatable and easy-to-define tasks)	**Non-routine** (Complex and difficult-to-define tasks)
**Manual** (Opportunities for automatization with robotics and computer vision)	• Monitoring of physiological parameters such as heart rate, blood pressure, respiration and body temperature • Timely and accurate administration of medication to patients • Disinfection and sterilization of medical equipment and facilities • Inpatient registration and management of insurance information	• Accurate and precise procedural skills for performing surgical interventions, such as incisions, sutures, and handling soft tissue or bone • Guidance of patients with physical therapy exercises • The operation of diagnostic imaging instruments, such as X-rays, computerized tomography, and magnetic resonance imaging equipment • Transportation of patients within a medical facility
**Cognitive** (Opportunities for automatization using machine learning and natural language processing)	• Charting and note-taking of patient history and physical examination • Pre- and intraoperative surgical planning • Individualized disease risk prediction and prognosis • Interpretation of clinical findings, lab results, and diagnostic imaging to guide patient management	• Synthesis of clinical practice guidelines based on the current state of evidence • Writing scientific research articles • Communication among and between various stakeholders, such as patients, insurance providers, and healthcare professionals (patient education, counseling) • Intuitive clinical and surgical decision-making

Adapted from Autor and Levy et al. [3]

## How can medical research benefit from the implementation of AI systems?

In recent years, the growing availability of healthcare data and the increasing maturity of AI as a technological tool initiated a gradual transformation of the medical research landscape. Patient registries containing granular information about the demographics and therapeutic interventions of numerous patient populations present new avenues for research in the age of big data. Electronic medical records permit the storage and traceability of data collected over the entire duration of medical treatment for patients with different medical conditions, including patient history, physical examination results, diagnostic images, interventions and outcomes over time.

Artificial intelligence has the potential to revolutionize medical research by enabling rapid and accurate analysis of vast amounts of data, containing demographic, genetic, clinical, surgical, and rehabilitation-specific information or a combination of these from thousands of patients, in pursuit of patterns associated with specific diseases or conditions. Furthermore, many AI systems possess the advantage of the ability to detect patterns, trends and connections that may not be easily recognized by humans, potentially leading to new clinical insights and breakthroughs in disease prevention, diagnostics and treatment. Analysis of large datasets, often with multimodal data content (in terms of the source and type of medical data), would be tedious and inefficient with the statistical methods currently employed in medical research [22]. Another benefit is that the automation of disease modeling, prediction, and diagnosis can potentially be performed online, with systems directly connected to relevant data sources and streams. Interconnectivity would enable the implementation of early warning signals to prompt further investigation and action by healthcare professionals when AI-intensive systems malfunction.

## A general overview of AI applications in medicine

Applications of AI can be useful in a broad range of research scenarios with far-reaching potential for clinical utility. The aim of this section is to provide the reader with a broad overview of areas with vast potential in orthopaedics using existing examples from AI-intensive medical research.

### Prediction of disease- or condition-specific outcomes

The continuous growth in the availability of high-quality medical data presents new avenues in the analysis of information derived from the results of clinical trials and national patient registries [2, 18]. One of the primary objectives of orthopaedic research is the primary and secondary prevention of musculoskeletal injuries and disease, and to identify operative or non-operative interventions that result in superior short- and long-term patient outcomes when disease and injury are already present. Machine learning (ML), which constitutes a subcategory of AI presents new opportunities in injury prevention and management through the identification of factors that predict a desired or undesired outcome [25]. As an example, ML-driven approaches may present new avenues for the prediction of reinjury risk in patients with anterior cruciate ligament reconstruction (ACL-R) given the availability of sufficient high-quality data. Such approaches need to account for the complexities of orthopaedic injuries by considering patient demographics, injury patterns, surgical factors and postoperative variables, including the quality and duration of rehabilitation before returning to preinjury activity levels. With expanding large-scale and multimodal orthopaedic datasets, the configurations of predictive variables and clinically important outcomes are unlimited, with vast benefits to both patients and healthcare professionals [47].

While the clinical implementation of AI-driven predictive algorithms is still in its nascency, their potential is demonstrated by several use cases in the current literature. One notable example is a clinical calculator for ACL revision risk prediction, developed with ML models applied to data from the Norwegian Knee Ligament Registry [27, 28]. While this project began to explore the application of a large volume of nationally collected patient data to improve injury risk management in orthopaedic sports medicine, additional studies are needed to determine the viability of registry data for the design of impactful predictive models harnessed in the everyday clinical setting [26].

### Medical image analysis

Image analysis is perhaps the most well-known application of AI in medicine. The ability of ML algorithms to perform classification and pattern recognition tasks when trained on radiographic images led to the proposal of numerous useful applications across fields, such as histopathology, dermatology, cardiology, ophthalmology, and radiology. Promising applications of AI and imaging technologies in these fields include the detection and grading of prostate cancer based on digitalized prostate biopsy slides [44], automated classification of benign and malignant skin lesions with dermatoscopic imaging [9], enhanced cardiovascular disease detection using electrocardiography [42], deep-learning enhanced detection of diabetic retinopathy and related diseases based on retinal images [45], and automated screening of chest radiographs for COVID-19 [36]. While the diagnostic ability of these systems is commendable, they currently show potential in augmenting expert clinical experience and decision-making, rather than altogether replacing the human component of diagnostic imaging.

In orthopaedics, AI-based image analysis applications have primarily made an impact on diagnostics, surgical planning, and implant design in traumatology, arthroplasty, and spine surgery. While similar approaches are currently underutilized in sports medicine, momentum is increasing in imaging applications for soft-tissue injury detection. A recent study demonstrated excellent diagnostic performance of an ACL tear detection ML algorithm trained on approximately 20,000 magnetic resonance images (MRI), with similar success after external validation on patient groups from two different countries [46]. Similarly, recent studies highlight the potential of automated meniscus tear detection, and rotator cuff segmentation using MRI data [16, 30]. Such studies demonstrate far reaching implications for the diagnosis of sports medicine injuries, but pathways for adoption into the everyday clinical workflow remain to be established. However, with rapid advances in areas like computer vision, surgical navigation, and video-analysis, the range of possibilities in orthopaedic sports medicine is only likely to expand.

### Decision support systems

The broad categories and types of data and ML models have led to advances in the implementation of multimodal AI systems [2]. Despite the widespread use of traditional rules-based decision support tools in the daily medical practice, AI-driven decision support systems promise to push the frontiers of evidence-based diagnosis, treatment, and clinical workflow. The overall aim of such systems is to improve the quality of care, individualize treatment, enhance patient outcomes, and simultaneously reduce complication and error rates in patient management. In orthopaedic trauma, there are a growing number of reports on the development of ML models for the detection and classification of fractures. However, only a small subset of the same studies evaluate the external validity of fracture detection tools, which hampers eventual adoption in the clinical setting [34]. Future studies should focus on improvement and assessment of the reliability of diagnostic, treatment-related, and prognostic models in the fields of orthopaedic trauma [34], spine surgery [29], sports medicine [38], and arthroplasty [7]. Advances are likely to result in the clinical application of integrated and robust AI-intensive decision support tools, with the potential to complement human expertise depending on the clinical context. It is noteworthy that the collaboration between humans and AI systems leads to superior performance compared with human experts and AI systems alone [6, 17, 40]. Future studies should aim to assess the influence of expertise level in orthopaedics (trainees, residents, fellows, experts) on the benefit of AI-assisted decision-making.

### Digital twin technology

Currently, evaluation of the efficiency and efficacy of medical interventions relies on time-consuming clinical trials, registry studies, and small-scale clinical investigations. While the results of clinical trials are considered the gold-standard of evidence synthesis, the clinical benefit of certain medical interventions may vary among individuals in a population. The digital twin is a concept adopted from engineering, and consists of a virtual representation of a real-world physical entity, such as an object, a system, or a patient [8]. The integration of high-quality multimodal data to design AI-driven digital twin models may enable real-time musculoskeletal injury prediction, assessment of the benefit of an orthopaedic intervention specific to an individual patient, simulation of surgical procedures, and evaluation of orthopaedic implant properties under various biomechanical conditions [2, 19]. As a proposed example, integration of virtual models of ACL-injured knees generated based on multimodal data from medical images, biomechanical tissue analyses, wearable sensors, and demographic information specific to individual patients may enable surgical planning and prognostics for orthopaedic interventions. Thus, “computational treatment” of a personalized knee model will provide information regarding the beneficial or harmful effects of the various treatment choices available in the real world. Additionally, digital twin technology can lead to new possibilities in the development of realistic arthroscopic knee surgery simulation training [33]. In the future, digital twins may facilitate individualized treatment across medicine and orthopaedics through real-time digital modeling of therapeutic intervention outcomes.

## Appraisal of quality and safety in medical AI research

The European AI Act, established in 2022, proposes a risk-based approach to the regulation of AI systems, and characterizes medical applications to be of high-risk [20]. Accordingly, the requirements proposed by the same European legal framework for safe implementation of high-risk AI systems include: 1) the use of high-quality datasets for training, testing, validation, and verification 2) thorough documentation of development to ensure traceability and auditability, 3) promotion of transparency and access to information by users, 4) measures allowing human oversight, 5) Accuracy, robustness, and adequate data security measures [20]. It is necessary to familiarize readers with several fundamental concepts for AI-intensive research projects to live up to these proposed criteria (Table 2).

**Table 2:** Definition of key terms for quality and safety in medical AI research

Term	Definition
Multimodal	In terms of health data, multimodality refers to the many distinct sources of data used by an AI system, such as electronic health records, medical imaging, wearable sensors, patient reported outcome measures, and others
Provenance	Thorough reporting of the origin and analysis of the analyzed data, model preparation, and model validation. The importance of documentation is paramount to ensure error detection, reliability, and reproducibility in AI-based research
Black box decision-making	Certain AI algorithms use methods for decision-making or predictive tasks that are uninterpretable to human observers. Black box models compromise the reliability and transparency of AI systems, as well as the potential for researchers to gain clinically relevant insights from such algorithms
Explainability	The possibility to trace how an AI system reached a conclusion in terms of input variables. Explainability is a key feature for error detection, bias elimination, and building trust in AI systems. Explainability also facilitates the inclusion of clinically relevant variables for model development
Distributional shift	Changes in the characteristics or patterns of the test population and biased training data may lead to decreased accuracy of an AI prediction system, as the model fails to adapt to unfamiliar data
Adversarial example	Data constructed different to the training examples, which may trick AI models to make incorrect predictions and jeopardize the safety of clinical prediction systems
Robustness	The proficiency of an AI system at handing real-world data, with large variations or deviation from the assumptions held by the trained models (missing data, outliers, adversarial examples)
Generalizability	The ability of AI systems to adapt to and correctly interpret data they were not trained on, thereby ensuring the elimination of hidden biases in datasets. Generalizable AI systems deliver consistent performance with patient groups that are adequately represented, as well as those underrepresented in the training data
Reproducibility	The ability of AI systems to produce consistent results when repeatedly trained on the same dataset
Replicability	The ability of AI systems to produce consistent results when repeatedly trained on different datasets
Uncertainty quantification	The process of measuring and determining the magnitude of uncertainty in the results of a predictive model based on input parameters, model characteristics, and inherent biases in the modeled system
Data labelling (annotation)	The task of identifying instances of relevant variables in a given dataset, such as predictors and outcomes, necessary to train models for the assessment of unlabeled test data

In the context of AI, provenance refers to the origins and history of a particular dataset or model. Provenance comprises information about how the data was collected, who collected it, where it was collected, and any transformations or modifications that were applied to it. Provenance is important in AI because it can help ensure that the data and models being used are reliable, trustworthy and can also help identify potential biases or errors in the data. Provenance in AI-based medical research is essential to build the trust required for clinical implementation of decision support systems and prediction tools, and to enable the design of replicable and transparent studies using AI. A hypothetical clinical decision support system designed to help clinicians optimize the treatment of patients with anterior cruciate ligament (ACL) injury can serve as an example to illustrate the role of provenance. Research studies for testing the validity of such a system will need to disclose the origin of the data the AI model was trained on, including the characteristics of the population, the types of variables collected, the timeframe of data collection, potential sources of bias, to name a few. Furthermore, the decision support system will require a detailed description of the data processing pipeline, model selection process, statistical analysis, methods applied to train, test, and validate models, as well as the parameters used to fine-tune the decision support system. Another important step is to disclose metrics used for the assessment of model performance. While seemingly a tedious task, ensuring provenance is necessary to meet the high standards required for the safe and reliable implementation of AI-driven medical research.

One of the major concerns with the ability of AI systems to predict events is that steps taken by certain models to reach predictions are often inaccessible. This characteristic, termed black-box decision making, results in an inability for human observers to explain model output in terms of the original input variables. This feature is particularly problematic for medical applications, as current decision making-systems are based on empirical rules, which allow human interpreters to trace the logic behind reasoning that leads to a certain outcome. This currently accepted and transparent approach enables humans to learn from systems, and perhaps as importantly, to detect and rectify errors and biases in the system, which may otherwise lead to false conclusions and even dangerous consequences. While methods have been proposed to improve the explainability of ML models, their implementation may not be feasible with all data types. Consequently, future AI-intensive medical research should focus on enhanced human interpretability, with the conversion of insight provided by the model to tangible knowledge that mirrors those of medical experts, with potential avenues for error detection. White-box ML models, aptly named to show the contrast to black-box models, provide a broken-down explanation of the steps taken to reach a conclusion with insights about how the input data was used throughout the decision process [24]. This feature is also the key to ensure that the evidence generated by AI-intensive orthopaedic research stems from correct representations of research problems with the available data, rather than potential anomalies or artifacts in datasets [24]. Explanations may vary depending on the type of data and ML model, and can consist of highlighted pixels on a medical image, highlighted text in written documents, relevance scores assigned to different variables used to make a prediction, and more abstract methods when necessary [32]. Essentially, a white-box approach to AI-intensive research answers how and why predictions are made and ensures that scientifically or clinically relevant building blocks are incorporated in the structure of models [39]. The immense value of such medical AI models lies in the capability to yield actionable insights to human users. Fundamental differences in interpretability between supervised and unsupervised learning approaches are beyond the scope of this section, and will be discussed in subsequent sections of this learning series.

As previously discussed, training AI systems on high-quality datasets is a major requirement for clinical adaptation. However, even models trained with the most attention to detail and with carefully curated data may not be universally applicable to every clinical setting. What happens when AI systems encounter unexpected changes in clinical context? Some examples of this phenomenon may be obvious, such as the erroneous prediction of ACL rerupture risk in female downhill skiers by a system that was trained predominantly on male football players. However, a more subtle example may be the poor reproducibility of ACL rerupture risk prediction in patients from one country based on a model trained on registry data from another, with different demographics, injury profiles, and surgical techniques. The inability of AI systems to adapt to new situations, termed distributional shift, is a central problem for the universal application of models across different settings, and may be influenced by countless forms of selection bias that are difficult for researchers to foresee [12]. Recent evaluation of generalizability in relation to the use of predictive models for acute kidney injury incidence in hospitalized patients revealed discrepancies in performance when applied to different patient populations [11]. The observed shortcomings in performance were attributed to the shortcoming that the acute kidney injury prediction models were trained on patient data collected from military veterans, and therefore failed to generalize to a more heterogeneous hospital patient cohort. To avoid such pitfalls, AI-intensive predictive model design should strive for the inclusion of training data representative of the population it was intended for, accounting for sex-imbalance, racial disparity, and age composition. In the future, adversarial examples, which serve to trick AI systems into making false predictions, may also be applied to check for model robustness, generalizability, and safety of use with data dissimilar to the training sample [21, 31]. Additionally, verification of reliable AI systems will likely be achieved though the assessment of reproducibility in performance with different training instances on the same dataset, as well as replicability of performance with training datasets that substantially differ in composition [31].

Recent developments towards standardizing the reports of AI-intensive research include the CONSORT-AI [23], STARD-AI [43], TRIPOD-AI [13], PROBAST-AI [13] and SPIRIT-AI [14] guidelines, which facilitate the implementation of rigorous guidelines reporting results and risk of bias in AI-intensive research. Notably, the DECIDE-AI [48] guideline was recently developed for the critical appraisal of studies reporting on early-stage, AI-driven clinical decision support systems. However, current guidelines primarily focus on the assessment of diagnostic interventions and clinical trials, which may only partially address the needs of the orthopaedic AI research community. The present learning series in AI-intensive research methods aims to address the increasing need for guidelines specific to AI-intensive orthopaedic research by culmination in a design and reporting checklist applicable across the broad range of study designs on the spectrum of evidence-based medical research.

## Barriers to implementation – threats and ethical considerations

While the deployment of AI systems opens exciting possibilities in medical research, mitigation of the potential risks of false predictions will be an essential task in the ensuing years. Navigation between models that produce truthful versus misleading outputs may present unique challenges. An important question is the role of human involvement in the training phase of models used in AI systems. While medical research is heavily rooted in evidence-based thinking and expert consensus, it is also prone to human error and bias. Consequently, excessive human supervision in AI-driven research may force AI systems to make errors akin to those made by human reasoning. However, it is also clear that black-box models preclude the explainability required for the implementation of AI systems in high-risk clinical settings [12]. While current AI applications in healthcare primarily rely on correlative ML models, long-term clinical applications in orthopaedics will likely require models capable of conveying causal relationships between input data and research outcomes [4]. This is especially important when the goal is to develop reliable AI systems for predicting outcomes for hypothetical patients and scenarios.

This presents an important dilemma with practical and philosophical implications. One approach to solving complex research questions is to entrust models built on ground truths founded on human clinical knowledge and existing evidence. The advantage of such supervised learning is that truths are derived using representations comprehensible to humans, which in turn allows human assessment for correctness. Alternatively, certain models are capable of a more intuitive approach, with ground truths based on implicitly derived knowledge by the model, without human supervision of the learning process. In turn, an unsupervised learning approach can provide the benefit of superior pattern recognition and complex, intuitive reasoning at the cost of human interpretability and assessment of the clinical relevance in the underlying logic. Future research will be required to reconcile supervised and unsupervised approaches in medical AI system development, and to ensure explainability and truthfulness [10].

While the recent application of large language models, such as ChatGPT by Open AI [15, 35] and Med-PaLM [41] to medical prompt-answering and reasoning demonstrate impressive capabilities, attention must be drawn to the need for thorough human evaluation of such applications, and potential threats before safe implementation. In general, the potential threats of AI systems are of crucial relevance in medical research and clinical practice, where room for error in diagnostic and therapeutic interventions is slim, with the potential for serious consequences. Today, the output from AI systems can be generated based on a range of training data, including but not limited to patient registries, national databases, wearable devices, and clinical trials. In turn, rigorous measures must be taken by experts to collect and curate data and avoid biased results that lead to harmful conclusions. Rigorous uncertainty quantification of medical AI models is paramount to determine the certainty with which models can be applied to personalize medical treatment in the everyday clinical setting. Due to the complexity of state-of-the art ML models, uncertainty testing exceeds traditional statistical error assessment, and relies on various methods to convert a single prediction to a distribution of predictions [1]. Additionally, a recent study reported that large language models can generate output convincing to experts and difficult to discern from human-generated information. Consequently, a significant portion of future research efforts regarding the adoption of medical AI systems should focus on effective ways to monitor model safety and reliability, as well as responsible ways to publish AI-generated results. To pioneer trustworthy applications in orthopaedics, guidelines and checklists should be developed for a range of research and clinical applications, warranting interdisciplinary collaboration among medical professionals and computer scientists with AI expertise.

## Conclusion

The boundaries of the safe and ethical use of AI in orthopaedic research remain to be determined. In the long-term, over-reliance on AI-driven algorithmic diagnosis, risk-prediction, and prognostics may erode the critical thinking skills considered so essential for clinical medical practice today. Similar to the broad range of industries and scientific domains, careful planning will likely be required to strike the appropriate balance between human- and AI-driven innovation in orthopaedics and sports medicine. While AI will likely exceed human performance in areas such as data analysis, pattern recognition, and decision-making, the goal of clinicians and researchers will be to identify and execute innovative AI-driven applications in medicine and thereby enhance the quality of patient care. The aim of subsequent parts of this learning series is to supply readers with the competence to design and implement AI-driven research projects through proficiency in the following topics:

1. A fundamental technical introduction to AI and ML for orthopaedic researchers, with a focus on the potential approaches to be used in medical research.
2. Familiarity with the current state of AI in medical research and understanding of the potential benefit conferred by AI in orthopaedics.
3. Approaching hypotheses and research questions in orthopaedic research using AI methods and requirements for interdisciplinary collaboration.
4. Data management for AI-driven orthopaedic research projects.
5. Understanding and interpreting the output of ML-models and AI systems.
6. End-product verification, safety in clinical use, and regulatory concerns.
7. A comprehensive checklist with regards to the previous principles to guide implementation of AI-driven research in orthopaedics.
